# Supplementary material for: Cost-effectiveness of a whole-area testing pilot of asymptomatic SARS-CoV-2 infections with lateral flow devices: a modelling and economic analysis study
Source: BMC Health Serv Res. 2022 Sep 22;22:1190. doi: 10.1186/s12913-022-08511-3 (PMC9502892; doi:10.1186/s12913-022-08511-3)
Supplement: Supplementary file 1 — Additional file 1: Supplementary materials [38, 39]. [file 12913_2022_8511_MOESM1_ESM.docx]

**Cost-effectiveness of a whole-area testing pilot of asymptomatic SARS-CoV-2 infections with lateral flow devices: A modelling and economic analysis study**

**Drakesmith et al**

**Supplementary material**

## Mathematical description of the model

The estimated number of estimated cases prevented on day $t$, denoted $x_{prev}(t)$, takes the form of an ordinary differential equation. The number of infections seeded on each day is computed differently for 1st generation infections $x_{seed}^{(1)}(t)$ and higher generation infections $x_{seed}^{(n)}(t)$.

$$x_{seed}^{(1)}(t+1)=x_{prev}^{(0)}(t) R(t)$$

$$x_{seed}^{(n)}(t+1)=(x_{prev}^{(1)}(t)+x_{prev}^{(n)}(t))((1-p_{sym})r_{asym}+p_{sym}p_{sym,inf})) R(t)$$

where $R(t)$ is the reproduction number, $p_{sym}$ is the proportion of cases that are symptomatic, and $r_{asym}$ is the relative infectability of asymptomatic cases relative to symptomatic cases. The number of asymptomatic cases arising from prevented cases, given by $x_{prev}(t)(1-p_{sym})$ is assumed to contribute to the next generation of seeded cases. Additionally, a smaller number of symptomatic cases will also contribute to the next generation of seeded cases. This is given by $x_{prev}(t)(p_{sym}p_{sym,inf})$, where $p_{sym,inf}$ is the proportion of symptomatic cases that contribute to new cases. $p_{sym}$ and $p_{sym,inf}$ are assumed to be constant.

The initial number of infections projected (the 0th generation infections), $x_{prev}^{(0)}(t)$) are given by the number of asymptomatic LFD tests with confirmatory positive PCR test result, $n_{LFDtest+PCR}(t)$, and with those no confirmatory PCR tests$n_{LFDtest}(t)$, the positivity prediction value $p_{PPV}$, the proportion estimated to be asymptomatic, given by $1-p_{sym}$, the proportion of symptomatic cases and still contributing to new infections $p_{sym,inf}$ and the relative infectability of asymptomatic cases $r_{asym}$.

$$x_{prev}^{(0)}(t)=(n_{LFDtest+PCR}(t)+n_{LFDtest}(t)p_{PPV})((1-p_{sym})r_{asym}+p_{sym}p_{sym,inf})$$

$x_{prev}(t)$ for other generations is computed from the convolution of $x_{seed}(t)$ with the log-normal model of the distribution of infections over time, $p_{inf}(t)=\mathrm{LognormalPDF}(t;\mu,\sigma^{2})$. For 1st generation infections, only the portion of the distribution from the median $e^{\mu}$ is included in the convolution.

$$x_{prev}^{(1)}(t)=\int_{0}^{T} x_{seed}^{(1)}(\tau)p_{inf}(t+e^{\mu}-\tau) d\tau$$

For nth generation infections, the whole distribution is included in the convolution.

$$x_{prev}^{(n)}(t)=\int_{0}^{T} x_{seed}^{(n)}(\tau)p_{inf}(t-\tau) d\tau$$

where $T$ is the length of the interval being analysed.

The total number of prevented cases for each day is then obtained from the sum of prevented 1st and nth generation infections.

$$x_{prev}(t)=x_{prev}^{(1)}(t)+x_{prev}^{(n)}(t)$$

## 2. Sensitivity analysis of the model

Some of the assumptions underlying the analysis (see: Box 1) have a high degree of uncertainty. All these parameters depend on to categorisation of symptomatic and asymptomatic cases. There are nuances in the definitional boundary between symptomatic and asymptomatic infection across studies [**32**], including separate categorisations of presymptomatic and paucisymptomatic cases which were not made in this analysis. These nuances make this set of parameters particularly challenging to quantify. The parameters of concern are:

### 1. The proportion of asymptomatic cases that become symptomatic (assumption 8).

This value of 0.44 was obtained from the follow up case-control study undertaken at the same time as the mass testing [**20**]. This estimate is chosen due to its highly specific to the geography and demography of the mass testing sample. However, this is quite a high estimate compared to other estimates in the literature. In the systematic review of [**38**], about three quarters of asymptomatic positive cases, never go on to develop symptoms. We therefore test against a lower best-case estimate of 0.25.

### 2. The proportion of symptomatic cases who contribute to further transmission (assumption 7).

This value of 0.2 was obtained from contact tracing data in the Merthyr Tydfil LA, cross referencing contacts and identifying which contacts originate from symptomatic positive cases. Again, this is the chosen estimate for the main analysis as it is highly specific to the geography and demography of the mass testing sample. However, incompleteness in contact reporting means this value is likely to be under-estimated. A cross-sectional survey in the UK by [**39**] indicate the proportion of symptomatic cases who fail to self-isolate is much higher (0.75). We therefore use this as a best-case alternative for this parameter.

### 3. The relative infectability of asymptomatic cases compared to symptomatic cases (assumption 2).

The value of 0.58 was obtained from the meta-analysis of [**19**]. However, this estimate has very large confidence intervals (0.34 - 0.99, with the upper bound treating the two groups as virtually equal in infectability. We do not have robust way of estimating this parameter in the mass testing sample, so we use the central estimate from this meta-analysis as the main parameter and the bounds of the confidence interval from this meta-analysis as best-case and worst-case estimates.

Three parameters were highlighted for sensitivity analysis and varied to produce worst-case and best-case scenarios for the number of cases prevented and associated healthcare outcomes (worst case being highest number of cases prevented). Absolute and relative changes and corresponding sensitivities were computed for the best-case and worst-case scenarios for each parameter individually, with the base scenario being that used in the main analysis. The parameters used for the best- and worst-case scenarios used in the main analysis are shown in Table S1.

Results of sensitivity analysis are shown in Table S2. The first two parameters tested (proportion of asymptomatic cases that become symptomatic and proportion of symptomatic cases who contribute to further transmission) produced small relative sensitivity values (magnitude below 1). The number of cases prevented when adjusting these values to less conservative estimates are 424 and 644, respectively (representing increases of 18% and 79% of the original estimate). However, sensitivity to the relative infectability of asymptomatic cases was very large (greater than 2). The number of estimated cases prevented in the less conservative scenario is 1067 (an increase of 196%). The model is therefore particularly sensitive to this parameter and so uncertainly in its estimation should be carefully considered when evaluating estimates produced by this model.

**Table S1: Parameters varied to produce best- and worst-case scenarios**

| Parameter description | Original scenario | Worst-case  scenario | Best-case  scenario |
| --- | --- | --- | --- |
| Proportion of asymptomatic cases who become symptomatic | 0.439 | 0.439 | 0.250 |
| Proportion of symptomatic cases who contribute to further transmission | 0.201 | 0.201 | 0.751 |
| Relative infectability of asymptomatic cases, compared to symptomatic cases | 0.580 | 0.340 | 0.990 |

Table S2: Results of sensitivity analysis

| Parameter description | Original parameter | New parameter | Absolute parameter change | Relative Parameter change | New output | Absolute output change | Relative output change | Absolute sensitivity | Relative sensitivity |
| --- | --- | --- | --- | --- | --- | --- | --- | --- | --- |
| Proportion of asymptomatic cases who become symptomatic | 0.439 | 0.250 | -0.189 | -0.431 | 424 | 64 | 0.178 | -338 | -0.412 |
| Proportion of symptomatic cases who contribute to further transmission | 0.201 | 0.751 | 0.550 | 2.735 | 644 | 284 | 0.789 | 516 | 0.288 |
| Relative infectability of asymptomatic cases, compared to symptomatic cases | 0.580 | 0.990 | 0.410 | 0.707 | 1067 | 707 | 1.964 | 1724 | 2.778 |

## 3. Sensitivity analysis of net monetary benefit estimates

The majority of benefits (around 90%) are through the discounted QALYs lost and costs of people dying from COVID-19. In addition to sensitive analysis of the model, we performed sensitivity analysis on the estimates of NMB (Table S3). We varied the QALYs lost from early deaths down from 6.78 down to 5 QALYs, but the net monetary benefits were still large at £4.6million. We also had a sensitivity analysis where we varied the value of QALYs down from £60,000 to £30,000 but the net monetary benefit was still positive at £3.0million. Using a 3.5% discount rate for health benefits (as recommended by NICE) instead of UK Treasury 1.5% slightly decreased the net monetary benefit from £6.2million to £5.7million. These net monetary benefits are from a health perspective and do not include wider economic benefits such as productivity gains.

Table S3: Results of sensitivity analysis of NMB estimates.

| Scenario | NMB (£) | 95% CI | |
| --- | --- | --- | --- |
|  |  | Lower | Upper |
| Original | 6,240,594 | 4,455,127 | 8,448,442 |
| Worst-case model parameters | 3,473,831 | 2,579,534 | 4,792,587 |
| Best-case model parameters | 15,866,328 | 12,296,102 | 20,546,837 |
| QALY loss of 5 instead of 6.78 for COVID deaths | 4,638,594 | 3,280,327 | 6,312,442 |
| Valuing QALYs at £30,000 instead of £60,000 | 3,004,703 | 2,060,970 | 4,162,814 |
| 3.5% discount rate instead of 1.5% | 5,745,594 | 4,092,127 | 7,788,442 |

##

## 4. Mapping Cost-effectiveness thresholds

In order to find approximate thresholds of cost-effectiveness, a set of simple simulations were carried out to across a space across four key parameters that affect the cost efficiency, in order to map cost-effectiveness across these parameters. LFD test results for each set of parameters were simulated. Prevalence, reproduction time (Rt), test sensitivity and test uptake. The number of asymptomatic individuals with a positive LTD test result is given by

$$n_{LFDtest}=Pn_{tested}(1-P_{sym})$$

where $P$ is the prevalence and $n_{tested}$ is the number tested, which is given by

$$n_{tested}=Np_{offered}p_{uptake}$$

Where $N$ is the total population, $p_{offered}$ is the proportion offered tests and $p_{uptake}$ is the proportion of uptake. $p_{offered}=0.9$. We assume that no confirmatory PCR tests are performed, and that number of true LFD positives is as given by the PPV value $p_{PPV}$, which was computed for the given test sensitivity and specificity values. Test specificity was fixed at 99%.

The simulated values of $n_{LFDtest}$ were uniformly distributed over a $T=30$ day period, so that $n_{LFDtest}(t)=n_{LFDtest}/T$. The simulated $n_{LFDtest}(t)$ and $p_{PPV}$ values were then fed into the model as described in supplementary materials 1. All other model parameters were as used in the original analysis. The same time-lagged model used in the main analysis was used to estimate hospitalisations and deaths prevented. The same operational costs, costs per test and QALYs from the main analysis was used to estimate ICER values.

The parameters ranges simulated were: Prevalence: [1%,2%,3%,4%,5%], Rt: [0.5,1.0,1.5,2.0,2.5], test sensitivity: [5%, 25%, 50%] and test uptake: [25%, 50%, 75%]. The simulated ICERs were mapped across the four parameters (Figure S1). ICERs were grouped into four levels of cost-efficiency: very high (<£0), high (£0-£15k), marginal (£15k-£30k) and none (>£30k). The approximate position of the mass testing pilot (Rt ≈ 1, prevalence ≈ 0.03, test sensitivity ≈ 50%, test uptake ≈ 50%) is indicated.

The overall trend of cost-effectiveness increases as prevalence and Rt increases. Cost-effectiveness shows a very high effect of test sensitivity, with a sensitivity of 5% making most of the parameter space non-cost-effective. Test uptake has more impact when test sensitivity low. At 50% sensitivity, there is only a small effect of test update.

With respect the conditions around the mass testing pilot, cost-effectiveness is predicted to be high or very high (ICER < £15k) when prevalence is above 2%, with an Rt of 1, or when Rt is above 0.75, with a prevalence of 3%.


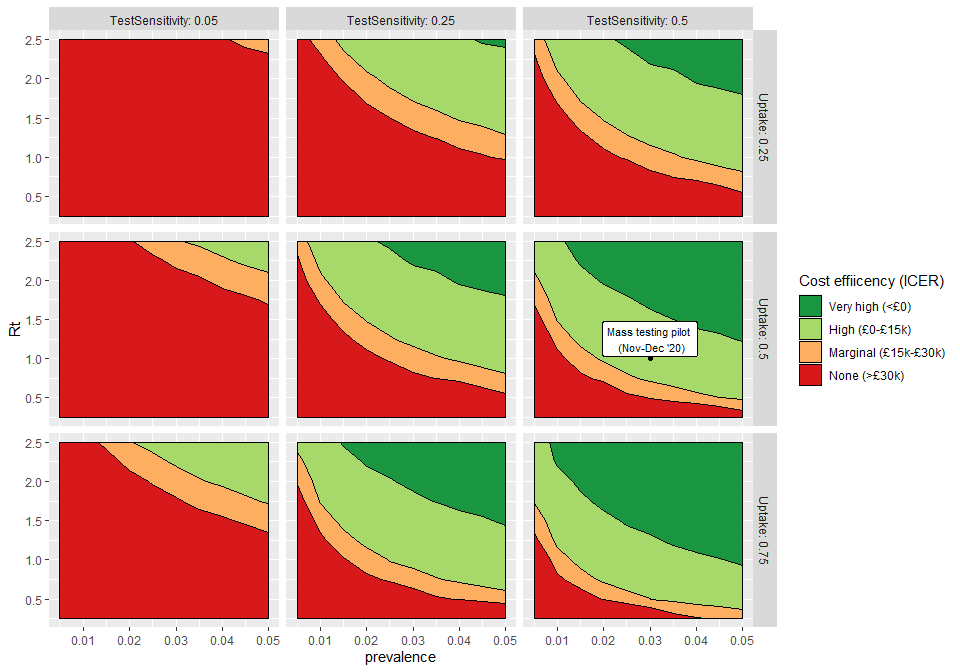


**Figure S1: Simulated ICER levels across four parameters: Rt, prevalence, test sensitivity and test uptake. All other model parameters fixed as defined in the main analysis. The approximate position of the mass testing pilot in the parameter space is indicated.**
